# A rise-assisting robot extends life space and improves facial expressions of nursing home residents

**DOI:** 10.1186/s12913-022-08952-w

**Published:** 2022-12-27

**Authors:** Kenji Kato, Tatsuya Yoshimi, Keita Aimoto, Kenji Sato, Naoki Itoh, Izumi Kondo

**Affiliations:** 1 Laboratory for Clinical Evaluation with Robotics, Assistive Robot Center, National Center for Geriatrics and Gerontology, 7-430, Morioka, Obu, Aichi 474-8511 Japan; 2 Department of Rehabilitation Medicine, National Center for Geriatrics and Gerontology, 7-430, Morioka, Obu, Aichi 474-8511 Japan; 3 Assistive Robot Center, National Center for Geriatrics and Gerontology, 7-430, Morioka, Obu, Aichi 474-8511 Japan

**Keywords:** Assistive robots, Nursing homes, Mobility, Life space, Facial expression

## Abstract

**Background:**

The introduction of nursing care-support devices using robotic technology is expected to reduce the task burden in long-term care facilities.

**Objective:**

To investigate the use of the rise-assisting robot, Resyone, in extending and improving the life space of nursing home residents with severe care needs.

**Methods:**

We performed a feasibility study in which Resyone was used to facilitate visits to additional sites in and around the nursing home as part of the care package of three residents. Two weeks before and four weeks after implementation of the new arrangements, the 30 caregivers involved were asked to record transfer times and destinations, while also checking the residents’ facial expressions.

**Results:**

Before implementation, participants had limited life spaces, but afterwards they regularly visited additional destinations including the garden, home entrance and corridors, which previously they had not visited frequently. The residents' facial expressions became more positive and less negative. This study demonstrates that Resyone can enrich care activities in severely disabled individuals.

**Conclusion:**

These findings suggest that the sustainable use of Resyone would improve the quality of care at care facilities. Moreover, the extension of otherwise limited life space has the potential to improve care receivers’ quality of life.

**Trial registration:**

UMIN Clinical Trials Registry No. UMIN000039204 (20/01/2020); retrospectively registered; interventional study; parallel, non-randomized, single blinded. URL of trial registry records: https://upload.umin.ac.jp/cgi-open-bin/ctr_e/ctr_view.cgi?recptno=R000044709.

**Supplementary Information:**

The online version contains supplementary material available at 10.1186/s12913-022-08952-w.

## Introduction

Recent advances in robotic technology have led to a growing interest in their use to support both patients and caregivers involved in nursing care [1–3]. Declining birthrates and aging populations, and the resulting shortage of caregivers, are a further stimulus to the use of assistive robots in nursing care worldwide [4–9]. The development of transfer support robots has been promoted because they can reduce strain on the lower back of caregivers, as described previously [9]. Another of our earlier studies showed that nursing homes have been sustainably using wearable transfer-assistive robots in a variety of direct care situations, such as transfer, toileting, and bathing assistances [8]. Other studies have suggested the use of a robotic lifting device [5] and a robot-assisted transfer device [10] to reduce the physical demand on caregivers during transfer care tasks.

The use of such transfer support robots is also highly desirable because they can improve the mobility and extend the life space of care-home residents. Life space can be thought of as the spatial extent of the environment in which an individual moves during a particular period of time, regardless of the type of activity or the use of mobility aids (e.g. wheelchair, walking-support robot) [11–13]. Previous studies on community-dwelling older adults have reported that life space is positively associated with a large number of individual as well as societal factors [14–16]. Nevertheless, there are few studies on the life space of nursing home residents [13, 17, 18]. Some studies have shown that residents spend most of their time lying or sitting down, which limits their mobility inside the nursing home [19, 20]. This reduction in life space and mobility can have negative consequences leading to drastic functional decline [21, 22]. In this context, it seems particularly important to explore the use of assistive robots to increase opportunities for transfers and to enlarge and enrich life space even for those residents who need a significant amount of support or care.

“Resyone” (“Resyone plus,” Panasonic AGE-FREE Co., Ltd., Osaka, Japan) is a rise-assisting robot that facilitates the transfer of bedridden residents who need severe care. It comprises half a care bed, which separates and transforms into an electric wheelchair [23, 24]. This separation is effected simply by pressing a switch; the only physical assistance required is the sideways movement of residents onto the relevant half of the bed. This device may not look like a robot as popularly conceived, but robotic technology is used in the safety system for combining and separating the wheelchair [23].

In a recent study, we introduced one Resyone unit to a nursing home resident who needed high levels of care, normally requiring two or three caregivers to assist with transfer tasks [9]. With continuous use of Resyone over 11 months, all transfer assistance could be performed by a single caregiver. Such improvements in caregiver efficiency with transfer tasks have a direct, beneficial effect on the care experience of residents, but are also likely to have indirect effects. For example, the ability of a single caregiver to provide transfer support means the additional caregivers that would have been involved are released to provide assistance with other care tasks elsewhere. Moreover, the increased flexibility provided by Resyone should allow residents to visit areas of the home that previously were beyond their capabilities, thereby extending their life space. However, no studies have yet been carried out to test this hypothesis, i.e. that assistive robots can extend a resident's life space.

The research question of our study is whether appropriate and sustainable use of robotic care devices may contribute to an improved quality of life. First, we prepared a feasibility study to extend residents’ life space by proposing new care operations to the caregivers using Resyone. We identified the existing living area for three residents and proposed additional care operations with Resyone such as: 1) going outside around the garden, 2) looking at the garden from the entrance, and 3) going around the corridor to look outside. These proposals were made after the caregivers had become familiar with the operation of Resyone and could perform transfer assistance tasks using only a single caregiver. Thus, the purpose of the present study was to investigate the feasibility of the additional care operations, with the assistance of a Resyone unit, over a period of four weeks. Furthermore, because the extent and quality of an individual’s life space is known to affect psychosocial conditions including depression [14, 25–27] and apathy [25] in older persons, we explored the effect of the above new care operations on quality of life by examining changes in the residents’ facial expressions associated with the ability to experience the environment beyond their private rooms.

## Methods

### Participants

In the present study, we chose a nursing home that where Resyone units had been in operation for at least 8 months after the first introduction of Resyone; it was identical to that in our recent report [9]. In this study, a total of three Resyone units were used with three residents (one male and two females, average age: 82.5 ± 5.0 years, weight: 45 ± 10 kg, height: 161 ± 11 cm) who required transfer assistance from multiple caregivers without Resyone. The basic characteristics, independence level, and length of time that individuals had used Resyone before the start of the present study are shown in Table 1.

**Table 1:** Demographics and independence level of the nursing home residents in this study

Subject ID	Length of time using Resyone (months)^a^	Gender	Age range	Level of disability^b^	Level of dementia^c^	Height range (cm)	Weight range (kg)
1	8	Male	80–84	C	IIIa	170–180	50–60
2	0	Female	75–79	C	IV	150–160	40–50
3	5	Female	85–89	C	M	150–160	< 40

*Footnote*: ^a^This is the time before the onset of the study. Daily life independence levels of older adults with disability^b^ and with dementia^c^ are defined by Ministry of Health, Labour and Welfare, Japan; explanations are provided in Supplementary Tables 1 and 2, respectively

For the male resident (ID1), the daily life independence level according to a scale for the older adult with disability (Supplementary Table 1) was Grade C, defined as “Remains in bed all the time, and requires care for toilet use, eating and changing clothes,” while the daily life independence level according to a scale for the older adult with dementia (Supplementary Table 2) was level IIIa, defined as “Symptoms, behavior or difficulty in communication that interfere with daily life.” Both female residents (ID2 and ID3) also showed a disability level of Grade C, but their dementia was more severe, corresponding to levels IV and M, respectively. Although the facial expressions of such individuals become less pronounced as cognitive functions decline, caregivers participating in their daily care are able to recognize the momentary changes that do occur. A total of 30 full-time caregivers were involved in the study. The basic demographics and experience levels of the caregivers are shown in Table 2. The home that we selected here was a special nursing home for older adults. In general, special nursing homes in Japan are for people needing long-term care of level 3 or higher according to the long-term care classification used in Japan [28]. By empirical investigations of Resyone user at several nursing homes, we also documented the activities of daily living (ADL) levels of those who might benefit from the device. Supplementary Table 3 shows the ADL levels of residents for whom the device is appropriate.

**Table 2:** Demographics of the caregivers in this study

	Number	Age	Height (cm)	Weight (kg)	Working experience (years)
Male	9	41 ± 9	170 ± 5	64 ± 8	11 ± 7
Female	21	49 ± 11	156 ± 6	56 ± 8	12 ± 6

### First pre-survey interview

Before the interventional study, two pre-survey interviews were held with five caregivers and the director and deputy director of the nursing home. The purpose of the first pre-survey interview was to check on the current situation regarding the use of Resyone, and to make suggestions for additional care operations with Resyone in the future.

**Fig. 1:**
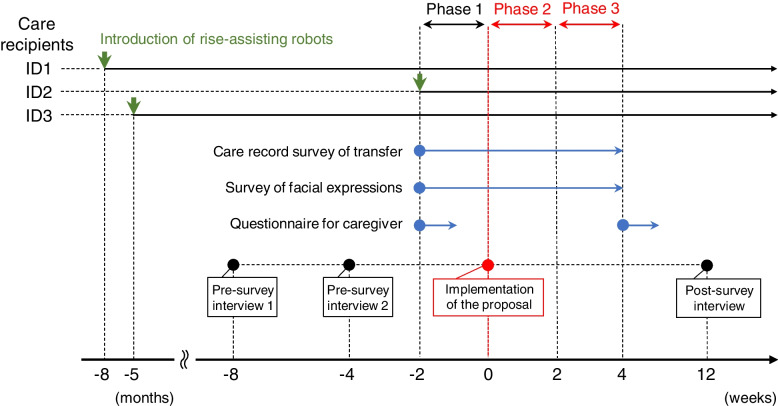
Timeline of the present research procedure. Green arrows indicate the introduction of Resyone for each resident (ID1, ID2 and ID3). The blue circles indicate the start points for the care record survey, the survey of facial expressions of the residents, and questionnaires for the caregivers. The red circle indicates the start point for implementing the proposed additional care operations. Black circles indicate when the pre-survey and post-survey interviews took place. Phases 1, 2 and 3 were set at two weeks each, with Phase 1 being prior to the introduction of additional care operations and Phases 2 and 3 being after their introduction

**Fig. 2:**
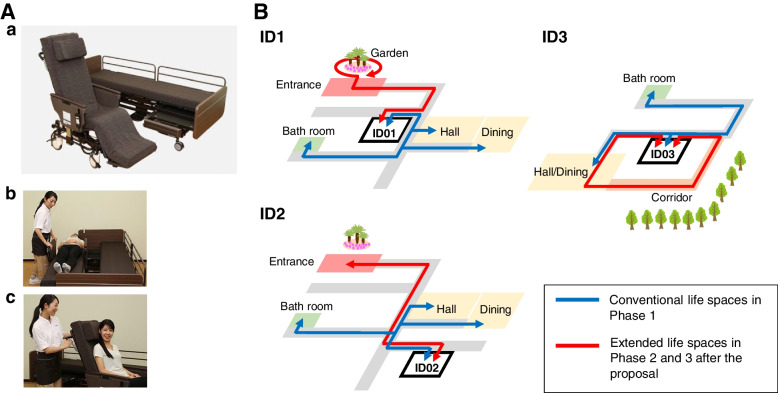
Schematic diagram of the nursing home involved in the care operations for the three residents**A** Overall view of Resyone (a) and images of Resyone separating into two parts (b) and functioning as a wheelchair (c)**B** The blue arrows indicated the living areas (conventional life spaces) that each resident visited from their private room in Phase 1. The red arrows indicate the additional routes resulting from the implementation of new care operations in Phases 2 and 3, which involved destinations that had not been visited before or were visited infrequently. Photographs used here are taken from our previous study [9]

First, we confirmed that the two residents (ID1 and ID3) who had been using Resyone were now able to transfer with only a single caregiver, whereas before the introduction of Resyone they had been assisted by two or three caregivers. ID1 was the same resident reported in the previous study [9]. Resident ID2 had not used Resyone before, but normally required transfer assistance from two or three caregivers. We concluded that the use of Resyone would be preferable for her and therefore a new Resyone unit was introduced and included in the present study (Fig. 1). Second, in the previous study [9] some caregivers told us that, when using Resyone, the need for only a single person for transfer tasks allowed them more flexibility in their care of residents. Therefore, we proposed additional care operations to extend the life space of the three residents using Resyone (Fig. 2A). Specifically, we asked the caregivers which places each resident visited regularly, and as a result identified new destinations that were infrequently or never visited prior to the study. We confirmed that, apart from their private rooms, the three residents regularly visited the dining room, hall (common area) and bathroom only. Therefore, places such as gardens, the home entrance or corridors were candidates for an extension of living space. Therefore, we suggested regular visits to such places as additional care operations. We also asked caregivers to reserve their views on whether this suggestion was feasible until the second pre-survey interview.

### Second pre-survey interview

The second pre-survey interview was conducted in a similar manner to the first pre-survey interview. First, we confirmed that the caregivers had a positive opinion of our proposal for additional care operations, as they judged it might improve the residents’ quality of life. We therefore discussed with the caregivers which new places to visit for each resident. Considering the location of the private room of each resident, it was decided that ID1 might pass through the front door and go outside to circle the garden, and that ID2 might visit the home entrance and look at the garden from there (Fig. 2B). Before the introduction of the additional care operations, ID3 used to eat lunch in her private room, then went to the hall/dining room after lunch. Therefore, we proposed modifying the care operation to include moving along the corridor to view the scenery outside after lunch (Fig. 2B). After further discussions, we explained the details of the research protocol, which is described in the study design section below, and confirmed our request to the caregivers regarding the survey. We also told them that the aim of the present study was to examine whether it was feasible for the caregivers to implement this proposal, but without any obligation to do so, and without placing an additional physical burden on them.

### Post-survey interview

The post-survey interview, involving five caregivers, the director and the deputy director, was held after all aspects of the study were complete. In this interview, we presented some of the results of the study, and opinions were sought mainly about the feasibility of the proposal to extend the life space of the three residents using Resyone, and what could and could not be done with Resyone. In addition, we asked directly for details of changes in the residents' facial expressions before and after the new care operations were implemented.

### Study design

The survey was conducted in three phases: Phase 1 comprised the two weeks before implementation of the proposed new activities, and was followed by Phases 2 and 3, each also lasting two weeks. The additional care operations, which aimed to extend the life spaces of the three residents, began at the start of Phase 2 (Fig. 1). It should take at least 2 months for all caregivers participating to become proficient in the operation of Resyone [9]. However, Resyone had already been implemented for 8 months when started the study in the nursing home.

First, to investigate the number of visits to each destination after transfer of the three residents, we asked the caregivers to provide the following information in a care record survey whenever a transfer assistance occurred using Resyone during the study period: 1) the time when the transfer assistance occurred, 2) all places visited by each caregiver after the transfer (e.g. Hall, Garden, Entrance, Dining room, Bathroom, Event, Meeting). ‘Event’ here mainly indicates any contact, either regular or sporadic, with the local community in the nursing home, and ‘Meeting’ mainly indicates visits with family members of the resident.

Second, a survey to check the facial expressions of each resident was requested alongside the care record survey. For the facial expressions survey, the caregivers were asked to rate the facial expressions of the residents as either 1) Positive, 2) Neutral or 3) Negative at the time they arrived at the final destination. These surveys of the facial expressions and care records of the residents were requested to be completed at all times when transfer assistance occurred over six consecutive weeks of Phases 1–3.

Third, the caregivers were asked to complete two questionnaires, one at the beginning of Phase 1 and another at the end of Phase 3. The questionnaire consisted of the following questions based on the previous study [9]: 1) Has the number of caregivers involved in the residents’ care been reduced (two-caregivers to one-caregiver, etc.)?, 2) Was the physical burden on the caregivers reduced by using the device? and 3) Was it possible to provide effective care by using the device?, and 4) Did the caregivers want to actively use the device to support the residents’ independence? The questions were answered using one of the following four options: “Agree,” “Agree somewhat,” “Disagree somewhat,” and “Disagree.” The questionnaire also asked for responses in a descriptive format about the advantages and disadvantages of operating Resyone. Detailed information is attached in the Supplementary Table 4 and Supplementary Figs1–3.

The study plan was reviewed and approved by the Ethics and Conflict of Interest Committee of the National Center for Geriatrics and Gerontology (acceptance no. 1293–4). The study protocol was registered and published at UMIN Clinical Trials Registry(No. UMIN000039204, 20/01/2020.

### Analysis

The data obtained from the present study were summarised and analysed with MATLAB (The MathWorks Inc., MA, USA). First, from the care record survey, the distribution of visits to each destination by each resident was calculated. Next, from the survey of the residents' facial expressions, the changes in the percentage of positive, neutral and negative facial expressions of each resident were calculated. In addition, to explore the trend in changes in the facial expression over Phases 1–3, we assigned a score of “-1” for negative, “0” for neutral and “1” for positive expressions, and the average of the facial expression scores for each visit was calculated for each resident over Phases 1–3. Because of the limited number of residents in the study and the fact that each caregiver visited a different destination, we investigated these changes in life space and facial expressions for each resident individually.

## Results

Although the timing of the introduction of Resyone differed for each resident (8 months, 5 months, and 2 weeks before implementation of the proposal for residents ID1, ID2 and ID3, respectively, as shown in Fig. 1, this proposal was made when almost all of the caregivers in the nursing home were familiar with the operation of Resyone. The target residents were chosen according to the recommended ADL levels described in Supplementary Table 3.

**Fig. 3:**
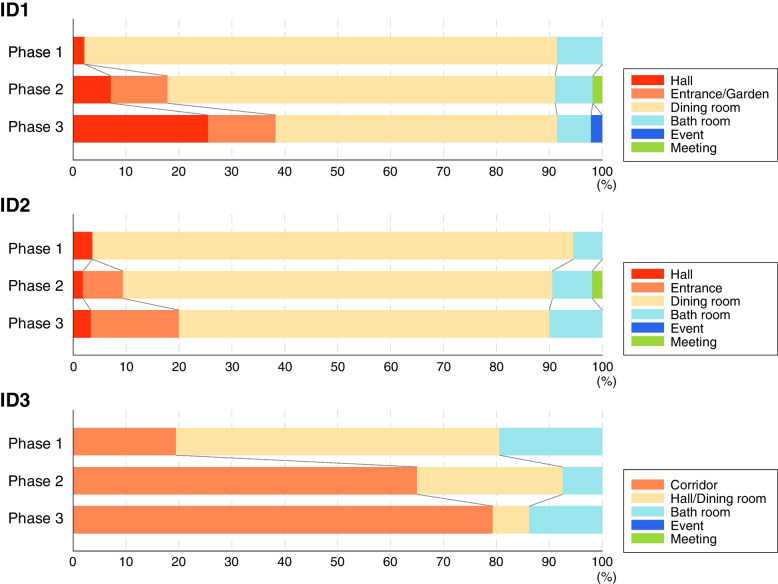
Distribution of destinations visited by the three residents using Resyone in Phases 1–3. All destinations visited by the residents during each phase of the study were counted and are shown as a percentage of the total number of visits in a bar graph format, with the destinations indicated in the key to each graph. The newly added care operations mainly included visits to the home entrance, garden or corridor; in Phases 2 and 3, the percentage of visits to those areas gradually increased for all residents

Using the data from the care record surveys, the number of transfers and the number of visits to each destination were summarized for the three residents over Phases 1–3 (Fig. 3). The results confirmed a gradual increase in the proportion of visits to newly targeted destinations for all three residents as the study progressed from Phase 1 to Phase 3. This is in line with the proposal that the additional care operations would encourage an extension of the residents’ life spaces (Fig. 2B). Specifically, for resident ID1, the new operation using Resyone to take a tour of the garden via the home entrance was added in Phases 2–3, resulting in the frequency of visits to the entrance and garden as well as the hall gradually increasing during Phases 2–3 (entrance and garden: 0% in Phase 1, 10% in Phase 2 and 12% in Phase 3; hall: 2% in Phase 1, 7% in Phase 2, 25% in Phase 3). A similar trend was observed for ID2, where Resyone was used to add the new operation of looking out from the home entrance (entrance: 0% in Phase 1, 7% in Phase 2, 16% in Phase 3). Similarly for ID3, for whom the new operation involved going around the corridor to look outside, access to the corridor was progressively more frequent over Phases 2–3 (corridor: 19% in Phase 1, 65% in Phase 2 and 79% in Phase 3). These results demonstrate that the new care operations using Resyone were able to extend the life spaces of the three residents, at least over the relevant four-week period of the study.

The results of the number of visits each resident made per day showed no significant changes over Phases 1–3. ID2 and ID3 showed a decreasing trend in Phase 3 (ID1: 47 visits in Phase 1, 58 visits in Phase 2, 45 visits in Phase 3; ID2: 55 times in Phase 1, 56 times in Phase 2, 27 times in Phase 3; ID3: 36 times in Phase 1, 42 times in Phase 2, 27 times in Phase 3). For ID2, the number of transfers decreased markedly in Phase 3. In the post-survey interview, we identified at least three days when ID2's physical condition deteriorated in Phase 3, which was likely a factor in the decreased number of transfers during this period.

The present study was carried out during the spread of SARS-CoV-2 (COVID-19 pandemic). We previously reported that, as a countermeasure implemented during the COVID-19 pandemic, meetings with family and friends were restricted entirely to communication through a glass shield at the entrance to the facility [6]. The post-survey interviews confirmed that access to the entrance room with Resyone in Phases 2–3 enabled ID1 to attend a meeting with family members in Phase 2 (green bar in Fig. 3) and a community meeting to celebrate longevity around the garden in Phase 3 (blue bar in Fig. 3). Similarly, ID2 was able to attend a meeting with a family member in Phase 2 (green bar in Fig. 3).

**Fig. 4:**
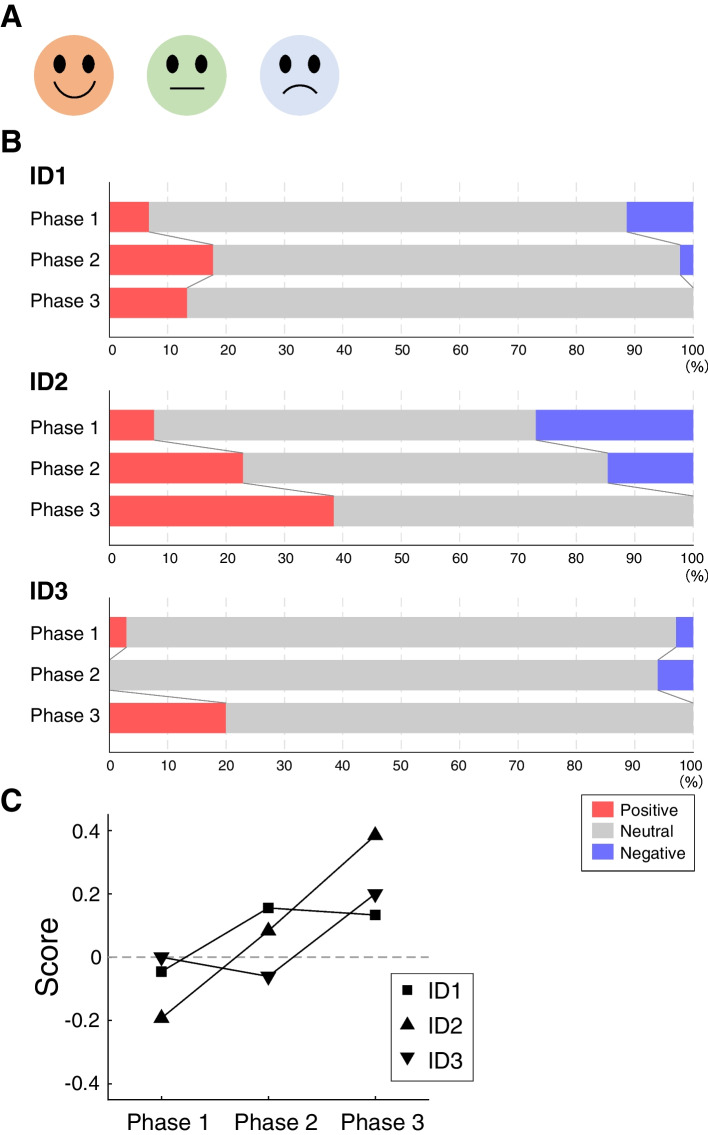
Changes in the residents’ facial expressions during progression from Phase 1 to Phase 3**A** The caregivers were asked to select the closest of the three facial expressions represented in the figure to those of the residents at each destination**B** Bar graphs of the percentage of facial expressions at each destination after transfer of residents in Phases 1–3. In each bar, the red, grey and blue colors represent positive, neutral and negative facial expressions, respectively**C** Changes in facial expression scores in Phases 1–3 in each resident

The facial expressions of each resident in Phases 1–3 on visiting each destination were investigated (Fig. 4). The proportion of positive facial expressions was higher in Phases 2 and 3 than in Phase 1 for ID1 (Phase 1; 6.8%; Phase 2; 17.8%; Phase 3; 13.3%) and ID2 (Phase 1; 7.7%; Phase 2; 22.9%; Phase 3; 38.5%), while the proportion of negative facial expressions was progressively lower in Phases 2 and 3 than in Phase 1 for ID1 (Phase 1; 11.4%; Phase 2; 2.2%; Phase 3; 0%) and ID2 (Phase 1; 26.9%; Phase 2; 14.6%; Phase 3; 0%). For ID3, there was little change in phase 2, but we observed an increase in positivity in phase 3 (Phase 1; 2.9%; Phase 2; 0%; Phase 3; 20.0%). For all residents, the proportion of negative facial expressions in Phase 3 was 0%. Next, these results were converted into scores with positive as 1, neutral as 0 and negative as -1. We found a gradual increase in scores over Phases 1–3 for all three residents (Fig. 4C).

Some of the responses collected from the questionnaires filled by the caregivers in Phases 1 and 3 are summarised in Supplementary Fig. 1. More than half of the caregivers responded positively (i.e. ‘Agree’ or ‘Agree somewhat’) to the first two questions, which query the opinions of caregivers. The results showed 70% (10% Agree, 60% Agree somewhat, 13% Disagree somewhat, 17% Disagree) of the responses were positive to the question “1) Has the number of people involved in the residents’ care been reduced (two-person to one-person, etc.)?” in Phase 1, and this increased to 84% in Phase 3 (13% Agree, 71% Agree somewhat, 10% Disagree somewhat, 6% Disagree). For the question “2) Was the physical burden reduced by using the device?” 66% (13% Agree, 53% Agree somewhat, 30% Disagree somewhat, 4% Disagree) of responses were positive in Phase 1, and 68% (16% Agree, 52% Agree somewhat, 23% Disagree somewhat, 9% Disagree) of responses were positive in Phase 3.

The two questions from the point of view of the residents also received more positive responses in Phase 3. The results showed that 50% (3% Agree, 47% Agree somewhat, 37% Disagree somewhat, 13% Disagree) of responses were positive for the question “3) Was it possible to provide effective care by using the device?” in Phase 1, and this increased to 61% (6% Agree, 55% Agree somewhat, 32% Disagree somewhat, 7% Disagree) in Phase 3. For the question “4) Did you (the resident) want to actively use the device to support your independence?” only 50% (13% Agree, 37% Agree somewhat, 40% Disagree somewhat, 10% Disagree) of responses were positive in Phase 1, and this slightly increased to 55% (13% Agree, 42% Agree somewhat, 35% Disagree somewhat, 10% Disagree) in Phase 3.

Lastly, other positive and negative points regarding the use of Resyone reported by the caregivers participated are listed as Supplementary Table 4, based on the free-text sections of the questionnaire and the opinions obtained from the post-survey interview of the caregivers. Supplementary Table 5 summarizes the problems that might occur when implementing Resyone in the chosen environment, together with appropriate countermeasures.

## Discussion

The present study demonstrated the ability of additional care operations to extend the life space of residents over a four-week period in a nursing home that was proficient in the use of Resyone rise-assisting devices. The three residents participating in the study were able to increase the frequency of visits to the garden, home entrance and corridor, which had been visited infrequently or not all previously. In Phases 2 and 3, we also confirmed an improvement in the residents' facial expressions at the destinations visited. These results support the idea that, even for residents with severe care needs, the long-term use of rise-assisting robots is an effective means of extending their life space and improving their quality of life.

To date, the commercially available transfer support robots such as Hug [5, 29], Resyone [6, 9], a wheelchair-mounted robotic assisted-transfer device [4, 10], and bathing assist device [30] have been reported to have the potential to reduce the physical load on caregivers in nursing homes. However, none of these has been discussed in the context of improving the mobility or life space of the residents, despite the fact that this is a primary aim of transfer tasks. In this sense, we believe that our findings are novel: even residents that are almost bedridden and have severe care needs are able to improve their life space through effective use of Resyone by a carer, mainly by extending life spaces beyond conventional areas (e.g. private room, bath and hall/dining) to other locations in the home (e.g. entrance or corridor) or outdoors (e.g. garden).

A previous study that used sensor-based assessment of life space in a nursing home showed that the majority of residents spend their time inactively, sitting or lying alone within the living unit, and very few individuals left their unit or facility [13]. Our findings were consistent with these, suggesting that nursing homes could be characterised as “barriers to social intercourse with the outside world,” as mentioned in Jansen et al., 2017 [13].

It should be noted that our study was conducted in a nursing home that accommodated residents who need assistive devices such as wheelchairs to move around and might need assistance to transfer to a wheelchair. Thus, there is a need for transfer assistance to ensure residents can access their life spaces, and, naturally, such access is heavily dependent on the availability of the caregivers, as well as other institutional factors [8, 13, 17], including care routines (timing and place of meals, timing and number of transfers) [13] and the scheduling of regular events [13, 17]. Other factors may include the constraints imposed by working environments in which caregivers ordinarily need to carry out a wide variety of care tasks with limited manpower, including direct care such as transferring, toileting, and mealtime and bathing assistance, as well as the documentation and administration of medication [8, 31, 32]. Considering these limitations, any reduction in the need for multiple caregivers for transfers as a result of using Resyone devices should contribute to an improvement in the operational efficiency of transfer assistance [9].

It was expected that the proposed additional care operations undertaken in this study would require some flexibility in the management of care tasks in the nursing home. Specifically, when visiting the garden or viewing the outside from the entrance with the resident, the caregiver was not able to predict in advance when they would return to the resident’s private room. In such cases, if multiple caregivers were needed for transfer assistance, it would be necessary to coordinate the caregivers’ schedules in advance or to wait for another caregiver to arrive. Clearly, therefore, it would be difficult to carry out such additional operations frequently and on a sustained basis. In this respect, our results suggest that the transformation from multiple- to single-caregiver assistance through the use of Resyone may lead to changes in the quality of care. This is consistent with some of the caregivers’ replies to the questionnaire, such as the ability “to flexibly change the time of transfer assistance,” “to perform assistance at whatever time the residents woke up.” On the other hand, there was no significant change in the number of transfers with Resyone for the three residents over Phases 1–3. This is probably due to the need for a certain level of care work and the physical load on the caregivers even with the use of Resyone.

Another finding of this study was that the extension of life space (Fig. 3) was related to positive changes in the residents’ facial expressions (Fig. 4). Previous studies have also reported that an individual's life space is associated with psychosocial factors in community-dwelling older adults [14, 26] and nursing home residents [25]. A remarkable result that emerged in the interview with the caregivers was that “One resident spoke for the first time ever” during one of the additional visits. Further details were obtained in the interview, which confirmed that this resident had subsequently continued to speak on other occasions. It seems reasonable to suppose that the novel visual stimuli the resident received at the new destination(s) provoked the changes in facial expression and conversational ability reported by the caregiver.

A previous study has suggested that a poor-quality relationship between patients with Alzheimer’s disease and their family caregivers is strongly associated with worse mental health in the caregivers [33]. Conversely, it was shown that the expression of genuine smiles by patients with dementia correlated with better mental health in family caregivers [34]. Therefore, what we observed may have a positive mental health effect not only on the resident, but also on the caregiver. As a future study, it would be interesting to investigate the long-term feasibility of the proposed care operations, as well as their psychological impact on residents and caregivers.

As a limitation of this study, it should be noted that the changes in the residents' facial expressions are qualitative data based on the caregivers' visual observations at the destinations and in the residents’ private rooms. However, the facial expressions were recorded by nearly 30 different caregivers, so individual bias was not likely. In recent years, novel artificial-intelligence-based emotional analysis tools that capture facial expressions have been validated in older people [35], and such tools could be applied in the future to provide a more quantitative analysis of psychological effects in a large scale study.

Finally, in the questionnaires in this study, we also identified some limitations of Resyone that arise due to interactions with the environment. Transfer-assistive robots are being developed worldwide, but in most cases their functionality is limited. More importantly, there are still many environmental or institutional factors that hinder the introduction of these assistive robots [36]. In the case of Resyone, some limitations were reported by caregivers, including the inability to climb steps (more than 5 mm), the difficulty of manoeuvring in a limited space such as a private room, and the heaviness (50 kg) of the wheelchair when moving around (summarized in Supplementary Table 5). Additionally we made a table of the target care recipients in terms of the ADL levels of those who might benefit from the device (Supplementary Table 3). Accordingly, it may be necessary to bear in mind the characteristics of each assistive robot when selecting users and to propose effective scenarios and care operations that are suited to the environment of the facility.

## Conclusion

The present study has demonstrated that certain care operations involving Resyone can promote life-space extension, even for residents with severe care needs. Based on this evidence, we hope that more nursing homes will utilize such assistive robots more effectively and sustainably, not only for the benefit of caregivers but also for the residents. We believe that the effective implementation and sustainable operation of assistive robots are pioneering the future of long-term care.

## Supplementary Information

**Additional file 1: Supplementary Table 1.** Criteria for determining the daily life independence level (bedridden level) of an older adult with disability **Supplementary Table 2.** Criteria for determining the daily life independence level of an older adult with dementia **Supplementary Table 3.** ADL levels of persons able to use Resyone, a transfer assistance device **Supplementary Table 4.** Responses in the free-text section of the questionnaire. **Supplementary Table 5.** Possible problems when implementing Resyone **Supplementary Figure 1.** The responses of caregivers to four questions from the questionnaire are shown. The responses increased in positivity (‘Agree’ and ‘Agree somewhat’), albeit only slightly, in Phase 3 compared to Phase 1 for thequestions 1) Has the number of caregivers involved in the residents’ care been reduced (two-caregivers to one-caregiver, etc.)?, 2) Was the physical burden on the caregivers reduced by using the device? and 3) Was it possible to provide effective care by using the device?, and 4) Did the caregivers want to actively use the device to support the residents’ independence? The questionnaire responses were obtained from 30 caregivers in Phase 1 and 31 in Phase 3, respectively **Supplementary Figure 2.** Survey of nursing staff attitudes (*before the proposal). **Supplementary Figure 3.** Survey of nursing staff attitudes (*after proposal, Last day of the empirical period).

## Data Availability

The datasets analyzed during the current study are not publicly available given that the research team has not completed its analysis, but are available from the corresponding author on reasonable request.

## References

[1] Bilyea A, Seth N, Nesathurai S, Abdullah HA (2017). Robotic assistants in personal care: a scoping review. Med Eng Phys.

[2] Kondo I (2019). Frailty in an aging society and the applications of robots. Japanese J Comp Rehab Sci.

[3] Lindeman DA, Kim KK, Gladstone C, Apesoa-Varano EC (2020). Technology and Caregiving: Emerging Interventions and Directions for Research. Gerontologist.

[4] Grindle GG, Wang H, Jeannis H, Teodorski E, Cooper RA (2015). Design and User Evaluation of a Wheelchair Mounted Robotic Assisted Transfer Device. Biomed Res Int.

[5] Yoshimi T, Kato K, Tsuchimoto S, Mizuguchi N, Kondo I (2021). Increase of verbal communication by long-term use of transfer-support robots in nursing facilities. Geriatr Gerontol Int.

[6] Yoshimi T, Kato K, Aimoto K, Sato K, Itoh N, Kondo I (2021). Utilization of transfer support equipment for meeting with family members in a nursing home during the COVID-19 pandemic: a case report. Geriatr Gerontol Int.

[7] Kowalski C, Arizpe-Gomez P, Fifelski C, Brinkmann A, Hein A (2020). Design of a supportive transfer robot system for caregivers to reduce physical strain during nursing activities. Stud Health Technol Inform..

[8] Kato K, Yoshimi T, Tsuchimoto S, Mizuguchi N, Aimoto K, Itoh N (2021). Identification of care tasks for the use of wearable transfer support robots – an observational study at nursing facilities using robots on a daily basis. BMC Health Serv Res.

[9] Kato K, Yoshimi T, Aimoto K, Sato K, Itoh N, Kondo I (2022). Reduction of multiple-caregiver assistance through the long-term use of a transfer support robot in a nursing facility. Assist Technol.

[10] Greenhalgh M, Blaauw E, Deepak N, St. Laurent COLM, Cooper R, Bendixen R (2020). Usability and task load comparison between a robotic assisted transfer device and a mechanical floor lift during caregiver assisted transfers on a care recipient. Disabil Rehabil Assist Technol.

[11] Stalvey BT, Owsley C, Sloane ME, Ball K (1999). The life space questionnaire: a measure of the extent of mobility of older adults. J Appl Gerontol.

[12] Tsai LT, Portegijs E, Rantakokko M, Viljanen A, Saajanaho M, Eronen J (2015). The association between objectively measured physical activity and life-space mobility among older people. Scand J Med Sci Sports.

[13] Jansen CP, Diegelmann M, Schnabel E-L, Wahl H-W, Hauer K (2017). Life-space and movement behavior in nursing home residents: results of a new sensor-based assessment and associated factors. BMC Geriatr.

[14] Parker M, Baker PS, Allman RM (2002). A life-space approach to functional assessment of mobility in the elderly. J Gerontol Soc Work.

[15] Webber SC, Porter MM, Menec VH (2010). Mobility in older adults: a comprehensive framework. Gerontologist.

[16] Taylor JK, Buchan IE, van der Veer SN (2019). Assessing life-space mobility for a more holistic view on wellbeing in geriatric research and clinical practice. Aging Clin Exp Res.

[17] Tinetti ME, Ginter SF (1990). The nursing home life-space diameter. J Am Geriatr Soc.

[18] Mortenson WB, Miller WC, Backman CL, Oliffe JL (2012). Association between mobility, participation, and wheelchair-related factors in long-term care residents who use wheelchairs as their primary means of mobility. J Am Geriatr Soc.

[19] Brown CJ, Redden DT, Flood KL, Allman RM (2009). The underrecognized epidemic of low mobility during hospitalization of older adults. J Am Geriatr Soc.

[20] den Ouden M, Bleijlevens MHC, Meijers JMM, Zwakhalen SMG, Braun SM, Tan FES (2015). Daily (in)activities of nursing home residents in their wards: an observation study. J Am Med Dir Assoc.

[21] Covinsky KE, Palmer RM, Fortinsky RH, Counsell SR, Stewart AL, Kresevic D (2003). Loss of independence in activities of daily living in older adults hospitalized with medical illnesses: increased vulnerability with age. J Am Geriatr Soc.

[22] Kortebein P, Symons TB, Ferrando A, Paddon-Jones D, Ronsen O, Protas E (2008). functional impact of 10 days of bed rest in healthy older adults. J Gerontol Series A.

[23] Kume Y, Tsukada S, Kawakami H (2015). Design and Evaluation of Rise Assisting Bed “Resyone®” based on ISO 13482. J Robot Soc Jpn.

[24] Kume Y, Tsukada S, Kawakami H (2019). Development of safety technology for rise assisting robot “Resyone Plus”. Trans JSME (in Japanese).

[25] Simmons SF, Schnelle JF, MacRae PG, Ouslander JG (1995). Wheelchairs as mobility restraints: predictors of wheelchair activity in Nonambulatory Nursing Home Residents. J Am Geriatr Soc.

[26] Tung JY, Rose RV, Gammada E, Lam I, Roy EA, Black SE (2014). Measuring life space in older adults with mild-to-moderate Alzheimer’s disease using mobile phone GPS. GER.

[27] Grace JM, Naiker J (2022). The association between objectively measured physical activity and health-related quality of life, life-space mobility and successful ageing in older Indian adults. Health SA Gesondheid (Online).

[28] Imahashi K, Kawagoe M, Eto F, Haga N (2007). Clinical status and dependency of the elderly requiring long-term care in Japan. Tohoku J Exp Med.

[29] Yoshimi T, Kato K, Tsuchimoto S, Mizuguchi N, Aimoto K, Itoh N, et al. Investigating proficiency using a lift-type transfer support device for effective care: comparison of skilled and unskilled nursing homes. Disabil Rehabil Assist Technol. 2022;0:1–10. 10.1080/17483107.2022.2128444 36178527

[30] Kato K, Aimoto K, Kawamura K, Yoshimi T, Itoh N, Kondo I. Novel bathing assist device decreases the physical burden on caregivers and difficulty of bathing activity in care recipients: a pilot study. Appl Sci. 2022;12:10131.

[31] Munyisia EN, Yu P, Hailey D (2011). How nursing staff spend their time on activities in a nursing home: an observational study. J Adv Nurs.

[32] Qian S-Y, Yu P, Zhang ZY, Hailey DM, Davy PJ, Nelson MI (2012). The work pattern of personal care workers in two Australian nursing homes: a time-motion study. BMC Health Serv Res.

[33] Mahoney R, Regan C, Katona C, Livingston G (2005). Anxiety and Depression in family caregivers of people with Alzheimer disease: the LASER-AD Study. Am J Geriatr Psychiatry.

[34] Lwi SJ, Casey JJ, Verstaen A, Connelly DE, Merrilees J, Levenson RW (2019). Genuine smiles by patients during marital interactions are associated with better caregiver mental health. J Gerontol Series B.

[35] Liu Y, Wang Z, Yu G (2021). The Effectiveness of Facial Expression Recognition in Detecting Emotional Responses to Sound Interventions in Older Adults With Dementia. Front Psychol..

[36] Servaty R, Kersten A, Brukamp K, Möhler R, Mueller M (2020). Implementation of robotic devices in nursing care. Barriers and facilitators: an integrative review. BMJ Open.

