# Supplementary material for: A rise-assisting robot extends life space and improves facial expressions of nursing home residents
Source: BMC Health Serv Res. 2022 Dec 27;22:1588. doi: 10.1186/s12913-022-08952-w (PMC9794109; doi:10.1186/s12913-022-08952-w)
Supplement: Supplementary file 1 — Additional file 1: Supplementary Table 1. Criteria for determining the daily life independence level (bedridden level) of an older adult with disability Supplementary Table 2. Criteria for determining the daily life independence level of an older adult with dementia Supplementary Table 3. ADL levels of persons able to use Resyone, a transfer assistance device Supplementary Table 4. Responses in the free-text section of the questionnaire. Supplementary Table 5. Possible problems when implementing Resyone Supplementary Figure 1. The responses of caregivers to four questions from the questionnaire are shown. The responses increased in positivity (‘Agree’ and ‘Agree somewhat’), albeit only slightly, in Phase 3 compared to Phase 1 for thequestions 1) Has the number of caregivers involved in the residents’ care been reduced (two-caregivers to one-caregiver, etc.)?, 2) Was the physical burden on the caregivers reduced by using the device? and 3) Was it possible to provide effective care by using the device?, and 4) Did the caregivers want to actively use the device to support the residents’ independence? The questionnaire responses were obtained from 30 caregivers in Phase 1 and 31 in Phase 3, respectively Supplementary Figure 2. Survey of nursing staff attitudes (*before the proposal). Supplementary Figure 3. Survey of nursing staff attitudes (*after proposal, Last day of the empirical period). [file 12913_2022_8952_MOESM1_ESM.pdf]

Supplementary Table 1.

Criteria for determining the daily life independence level (bedridden level) of an older adult with disability

**Grade J**	Has some sort of disability, but is almost independent in daily life and can get out of the home without assistance
(Independent living)	1. Can use transport facilities
	2. Can visit neighbors
**Grade A**	Almost independent for indoor daily life, but cannot go outside without assistance
(Requires assistance to leave home)	1. Goes out with assistance, and is out of bed for most of the daytime
	2. Does not go out much, and needs bedrest during the daytime
**Grade B**	Requires a degree of care for indoor daily life, and remains in bed most of the time, but can maintain a sitting position
(Nearly bedridden)	1. Eats and visits the toilet away from the bed using a wheelchair
	2. Requires assistance to use a wheelchair
**Grade C**	Remains in bed all of the time, and requires care for toilet use, eating, and changing clothes
(Completely bedridden)	1. Able to roll over without help
	2. Unable to roll over without help

Footnote: the grades and criteria are defined by Ministry of Health, Labour and Welfare, Japan. Available from: https://www.mhlw.go.jp/file/06-Seisakujouhou-12300000-Roukenkyoku/0000077382.pdf, https://www.mhlw.go.jp/english/database/db-hss/dl/siel-2010-04.pdf

Supplementary Table 2.

Criteria for determining the daily life independence level of an older adult with dementia

Grade		Assessment criteria	Examples of symptoms and behaviors that may be observed
I		Has some sort of dementia, but is almost independent in daily life for domestic and social activities	
II		Symptoms, behavior, or difficulty in communication that interfere with their daily life are observed to some degree, but can live independently if assisted by a carer	
	IIa	The condition described in II is observed outside the home	Frequently gets lost and/or makes mistakes with activities where they were previously competent such as shopping, paperwork, managing finances, etc.
	IIb	The condition described in II is also observed at home	Inability to manage medication and/or stay at home alone due to difficulties in answering the phone, dealing with visitors, etc.
III		Symptoms, behavior, or difficulty in communication that interfere with their daily life are observed occasionally, and requires care	
	IIIa	The conditions described in III are observed mainly during the day	Has difficulty and/or takes time to change clothes, eat, defecate, and/or urinate. Repeatedly puts things in their mouth, picks up things, wanders, becomes incontinent, shouts or screams, mismanages fire, has poor personal hygiene, exhibits sexually abnormal behavior, etc.
	IIIb	The conditions described in III are observed mainly at night	Same as level IIIa
IV		Symptoms, behavior, or difficulty in communication that interfere with their daily life are observed frequently, and requires constant care	Same as level III
M		Significant psychotic manifestations, problem behavior, or severe physical diseases are observed, and requires specialized medical care	Persistent psychiatric symptoms such as delirium, delusions, agitation, or self-harm or can harm others; problematic behavior caused by psychiatric symptoms

Footnote: the grades and criteria are defined by Ministry of Health, Labour and Welfare, Japan.

Available from: https://www.mhlw.go.jp/topics/2013/02/dl/tp0215-11-11d.pdf

Supplementary Table 3.

ADL levels of persons able to use Resyone, a transfer assistance device.

		Criteria for older adult with disability *1							
		**Grade J**		**Grade A**		**Grade B**		**Grade C**	
		**Independent**		**Requires assistance to leave home**		**Nearly bedridden**		**Bedridden**	
		**J1**	**J2**	**A1**	**A2**	**B1**	**B2**	**C1**	**C2**
**ADL levels**	**Sitting position**	Stable		Stable	Stable	Stable	Unstable	Impossible	
	**Standing (hold)**	Stable		Stable	Stable	Unstable	Impossible	Impossible	
	**Standing up**	Stable		Stable	Unstable	Unstable	Impossible	Impossible	
	**Application**	-		-	-	△	○	○	

Notes: ○, applicable: △, possible in some cases; ×, not applicable; -, no need to use; *1: see "Criteria for determining the daily life independence level (bedridden level) of an older adult with disability," described in **Supplementary Table 1**.

Supplementary Table 4. *Responses in the free-text section of the questionnaire.*

Classification	Responses	Number of responses
Positive	The introduction of Resyone enabled single-caregiver transfer assistance, which made it possible to be flexible about when transfer assistance tasks were performed and allowed the addition of new care operations.	8
Positive	The elimination of two-person assistance also eliminated the need for special schedules between caregivers. As a result, the range of activities of the resident was extended.	2
Positive	The use of Resyone enabled the caregiver to perform assistance at whatever time residents woke up, without having to wait for other caregivers.	1
Positive	The residents' faces relaxed and a smile appeared on their faces when they were able to move to the garden or to the corridor.	At the interview
Positive	It would have been difficult to change or add the proposed care operations without Resyone, as it would have required more than one caregiver to assist with transfers.	At the interview
Positive	One resident spoke for the first time ever (although we couldn't understand what she was talking about).	At the interview
Positive	The caregivers were able to stop briefly in the corridor to look out of the windows and have a conversation with the resident.	At the interview
Positive	No additional physical burden was felt in Phases 2 and 3.	At the interview
Negative	Manoeuvring Resyone in the private room was difficult due to the small space available.	8
Negative	I felt that Resyone was heavy (around 50 kg including battery) and more difficult to move than a normal wheelchair.	5
Negative	There was a concern about falling because the Resyone bed could not be lowered as low as a conventional nursing bed, i.e. more than 45 cm off the ground.	3
Negative	In the case of residents who were almost bedridden and their eyes mostly closed, it was sometimes difficult to perceive any changes during the additional care operations with Resyone.	2
Negative	When the caregivers used Resyone as a wheelchair for a long distance, the body of the residents gradually shifted forward and we had to adjust their posture accordingly.	2

Negative	Steps in the care home sometimes restricted mobility with Resyone, due to the small tire size.	1

Supplementary Table 5. *Possible problems when implementing Resyone.*

	Problem	Counter-measure
Infrastructure	Difficulty manoeuvring if the room is small	Move residents to larger rooms
	Difficulty climbing even small steps (<5mm)	Eliminate steps
Device	Heavy (50 kg as wheelchair)	Avoid carpeted floors
	Residents eventually slip down while seated in the wheelchair	Care must be taken to monitor the resident's position when using the device for a long time
	Increased fall potential due to high bed height (>45 cm)	Place a cushion pad under the bed (or do not use the device)

Supplementary Figure 1

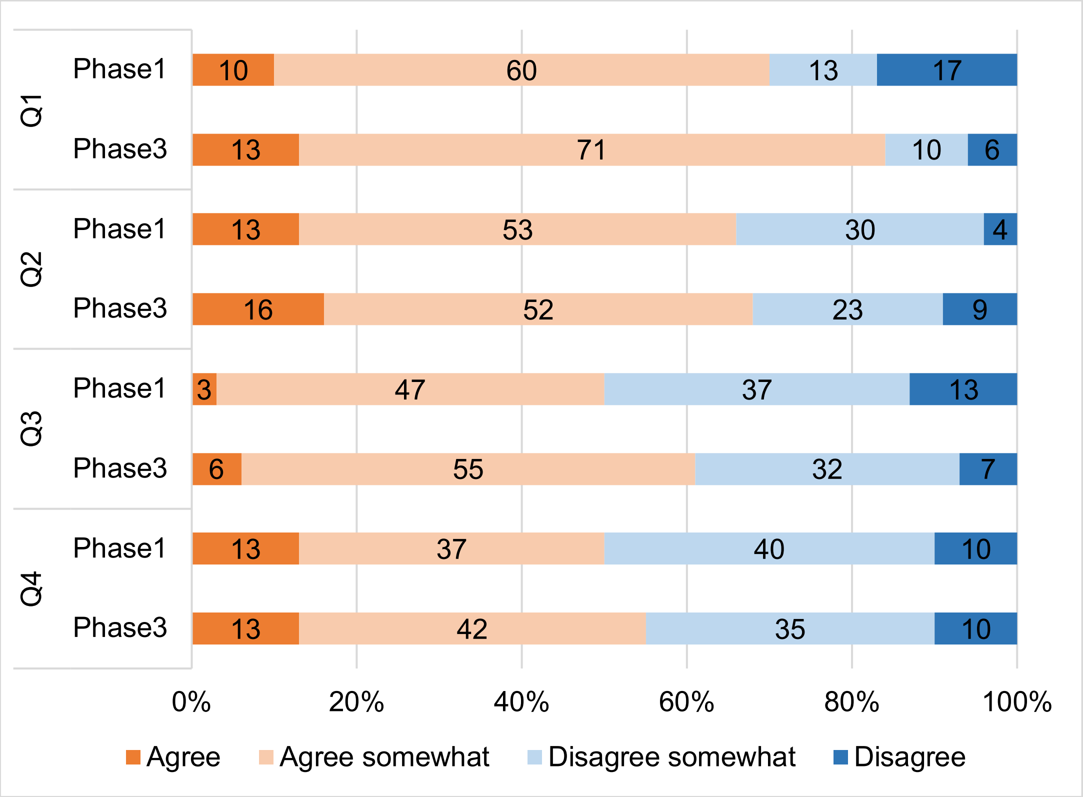

Supplementary Figure 1. The responses of caregivers to four questions from the questionnaire are shown. The responses increased in positivity ('Agree' and 'Agree somewhat'), albeit only slightly, in Phase 3 compared to Phase 1 for the questions 1) Has the number of caregivers involved in the residents' care been reduced (two-caregivers to one-caregiver, etc.)?, 2) Was the physical burden on the caregivers reduced by using the device? and 3) Was it possible to provide effective care by using the device?, and 4) Did the caregivers want to actively use the device to support the residents' independence? The questionnaire responses were obtained from 30 caregivers in Phase 1 and 31 in Phase 3, respectively.

Supplementary Figure 2.

【2】-1 Survey of nursing staff attitudes (*before the proposal)

Staff ID		Date filled in	
		MM	DD

Properties (Please circle all that apply)			
Sex	M ・ F	Age Range	10≤ ・ 20≤ ・ 30≤ ・ 40≤ ・ 50≤ ・ 60≤ ・ 70≤～
Height	<150㎝ ・ 150≤ ・ 160≤ ・ 170≤ ・ 180cm≤		
Weight	<40kg ・ 40≤ ・ 50≤ ・ 60≤ ・ 70≤ ・ 80kg≤		

Qualifications held (Please answer only if you are a care worker)			
Qualifications	(1) Helper Level 1, (2) Helper Level 2, (3) Care Worker, (4) Social Worker, (5) Public Health Nurse, (6) Nurses, (7) Licensed Practical Nurses, (8) Physical Therapists, (9) Occupational Therapists, (10) Speech Therapists, (11) Counselor for welfare equipment, (12) Prosthetician	Years of experience	y M
		Experience using Resyone	M

How do you feel about the device we are installing? (Please circle the answer that best describes)

1) Impact on caregivers using the device	Agree	Agree somewhat	Disagree somewhat	Disagree
A Do you expect that the use of Resyone will reduce physical burden?	1	2	3	4
B Do you expect to have sufficient mental capacity when performing transfers using Resyone?	1	2	3	4
C Do you expect that communication with the care recipient will be increased by the use of Resyone?	1	2	3	4
**2) Impact on the target care recipient using the device**	**Agree**	**Agree somewhat**	**Disagree somewhat**	**Disagree**
A More secure than human care	1	2	3	4
B Worried about safety	1	2	3	4
C Worried about hygiene	1	2	3	4
**3) Impact on care work**	**Agree**	**Agree somewhat**	**Disagree somewhat**	**Disagree**
A Increased the liveliness of the workplace	1	2	3	4
B The number of people involved in the care has been reduced (two-person → one-person, etc.)	1	2	3	4
C Efficient care time (increased other care time)	1	2	3	4
D Care was not streamlined because it took time to operate and manage the devi	1	2	3	4
**4) Thoughts arising when using the device**	**Agree**	**Agree somewhat**	**Disagree somewhat**	**Disagree**
A Will be able to provide effective care by using the equipment.	1	2	3	4
B Don't want to use the device because it is difficult to use, heavy and difficult to	1	2	3	4
C For independence support, it is important to be able to assist without using dev	1	2	3	4
D Want to actively use the equipment to support independence	1	2	3	4

Supplementary Figure 3.

【2】-2 Survey of nursing staff attitudes (*after proposal, Last day of the empirical period)

Staff ID	Date filled in
	MM DD

1. How do you feel about the device you have installed? (Please circle the answer that best applies to you)				
1) Impact on caregivers using the device	Agree	Agree somewhat	Disagree somewhat	Disagree
A Was physical burden reduced by the use of the device?	1	2	3	4
B Did you have sufficient mental capacity when performing transfers using the device?	1	2	3	4
C Was communication with the care recipient increased by the use of the device?	1	2	3	4
2) Impact on the target care recipient using the device	Agree	Agree somewhat	Disagree somewhat	Disagree
A Increased range of activities for residents using the device	1	2	3	4
B Increased social participation of individuals using Resyone	1	2	3	4
C Increased verbal communication by indivisuals using Resyone	1	2	3	4
D Changes in the facial expression of indivisuals using Resyone (e.g. more smiles)	1	2	3	4
3) Impact on care work	Agree	Agree somewhat	Disagree somewhat	Disagree
A Increased the liveliness of the workplace	1	2	3	4
B The number of people involved in the care has been reduced (two-person → one-person, etc.)	1	2	3	4
C Efficient care time (increased other care time)	1	2	3	4
D Care was not streamlined because it took time to operate and manage the device.	1	2	3	4
4) Thoughts arising when using the device	Agree	Agree somewhat	Disagree somewhat	Disagree
A Will be able to provide effective care by using the equipment.	1	2	3	4
B Don't want to use the device because it is heavy and difficult to use	1	2	3	4
C For independence support, it is important to be able to assist without using device	1	2	3	4
D Want to actively use the equipment to support independence	1	2	3	4

(2) What did you think of Resyone? Please let us know if your opinions.

If the range of activities of the user could be increased by using Resyone, please explain why; if not, please explain why not, from the viewpoint of the facility / staff, the user, the device (Resyone). Please answer freely for each reason as illustrated in the following examples.

	Reason that activities could be increased	Reason that activities could not be increased or only with difficulty
Facility / staff	(example)<br>・Even employees with back pain do not have to worry about transfer movements.<br>・The number of two-person caregiving has decreased, making transfers easier.<br>________<br>________	(example)<br>・There is a step to get out to the balcony, and it is difficult to cross the step with Resyone.<br>・It is difficult to change nursing care operations such as diaper changing time.<br>________<br>________
The user	(example)<br>・Rishone does not require transfer support to a wheelchair and makes transfer and movement easier.<br>・With Resyone, users can move to their destination without difficulty.<br>________<br>________	(example)<br>・Being almost bedridden makes it difficult for users to move with Resyone (other than eating and bathing) in the first place.<br>・Users prefer to stay in their living rooms<br>________<br>________
The device (Resyone)	(example)<br>・Because the bed becomes a wheelchair, transfer support is not required and user movility is increased.<br>・It would be more convenient if there were many Resyone units in the care home.<br>________<br>________	(example)<br>・It is not as easy to handle as a normal wheelchair.<br>・It is not suitable for outdoor use because the tires will get dirty.<br>・Difficult to use both indoors and outdoors<br>________<br>________
